# Do different anesthesia regimes affect hippocampal apoptosis and neurologic deficits in a rodent cardiac arrest model?

**DOI:** 10.1186/1471-2253-15-2

**Published:** 2015-01-15

**Authors:** Stepani Bendel, Dirk Springe, Adriano Pereira, Denis Grandgirard, Stephen L Leib, Alessandro Putzu, Jannis Schlickeiser, Stephan M Jakob, Jukka Takala, Matthias Haenggi

**Affiliations:** Department of Intensive Care Medicine, University Hospital – Inselspital and University of Bern, Bern, Switzerland; Department of Intensive Care Medicine, Kuopio University Hospital, Kuopio, Finland; Neuroinfection Laboratory, Institute for Infectious Diseases, University of Bern, Bern, Switzerland; Biology Division, Spiez Laboratory, Federal Office for Civil Protection, Spiez, Switzerland

**Keywords:** Cardiac arrest, Rats, Sevoflurane, Fentanyl, Ketamine, Medetomidine, Neuroprotection

## Abstract

**Background:**

Different anesthesia regimes are commonly used in experimental models of cardiac arrest, but the effects of various anesthetics on clinical outcome parameters are unknown. We conducted a study in which we subjected rats to cardiac arrest under medetomidine/ketamine or sevoflurane/fentanyl anesthesia.

**Methods:**

Asystolic cardiac arrest for 8 minutes was induced in 73 rats with a mixture of potassium chloride and esmolol. Daily behavioral and neurological examination included the open field test (OFT), the tape removal test (TRT) and a neurodeficit score (NDS). Animals were randomized for sacrifice on day 2 or day 5 and brains were harvested for histology in the hippocampus cornus ammonis segment CA1. The inflammatory markers IL-6, TNF-α, MCP-1 and MIP-1α were assessed in cerebrospinal fluid (CSF). Proportions of survival were tested with the Fisher’s exact test, repeated measurements were assessed with the Friedman’s test; the baseline values were tested using Mann–Whitney *U* test and the difference of results of repeated measures were compared.

**Results:**

In 31 animals that survived beyond 24 hours neither OFT, TRT nor NDS differed between the groups; histology was similar on day 2. On day 5, significantly more apoptosis in the CA1 segment of the hippocampus was found in the sevoflurane/fentanyl group. MCP-1 was higher on day 5 in the sevoflurane/fentanyl group (p = 0.04). All other cyto- and chemokines were below detection threshold.

**Conclusion:**

In our cardiac arrest model neurological function was not influenced by different anesthetic regimes; in contrast, anesthesia with sevoflurane/fentanyl results in increased CSF inflammation and histologic damage at day 5 post cardiac arrest.

## Background

Sudden cardiac arrest (CA) is the most important cause of global cerebral ischemia. Only a minority of resuscitated patients are discharged in good neurological condition [1]. After CA, the most relevant factor contributing to recovery is the time interval between collapse and initiation of resuscitation efforts and the return of spontaneous circulation (ROSC). Despite community wide efforts to improve the chain of survival, most patients will not regain consciousness after ROSC, indicating some degree of neuronal injury after transient ischemia. However, neuronal injury is not uniformly distributed, and follows a time course [2]. Vulnerable brain areas (thalamic reticular nucleus, hippocampal cornus ammonis sector, caudate nucleus and cerebellar Purkinje cells) demonstrate delayed neuronal degeneration with characteristic signs of apoptosis [2]. This delayed neuronal degeneration theoretically opens a window of opportunity to mitigate the devastating effects of ischemia on the brain. Despite multiple efforts to alter this pathway, the only neuroprotective measure that has profound effect on survival and functional outcome after CA is hypothermic [3, 4] or normothermic [5] targeted temperature control.

Different rodent CA models have been developed, with induction of CA of different duration by either ventricular fibrillation (6–12 minutes) [6, 7], asphyxiation (6–12 minutes) [8], hypothermia (30 min) [9], injection of potassium chloride or other cardioplegic agents (5–6 min) [10]. Whereas all these models simulate the same global brain ischemia, the anesthesia methods differ mainly in two ways: use of intraperitoneal (IP) barbiturates [6, 9] or anesthetic vapors [7, 9, 10]. Both barbiturates and volatile anesthetics have, at least to some degree, neuroprotective properties [11], thus potentially influencing the results of experimental CA models. Most comatose survivors of cardiac arrest in the intensive care unit need sedative drugs to facilitate mechanical ventilation and reduce sympaticotonic vegetative burden. No formal guidelines exist for this clinical situation, so various regimes are used [12]. Due to technical limitations, vapor anesthetics have been introduced only lately in this patient group [13].

During implementation of a rodent CA model in our lab, we evaluated the effects of two different anesthetic regimes on brain damage, defined by brain histology and inflammatory markers in the cerebrospinal fluid, and on neurologic deficits, examined by behavioral tests. We choose the volatile anesthetic sevoflurane in combination with fentanyl, which is considered displaying at least some neuroprotective properties [11]. The comparator was a standard rodent anesthesia regime with the α-agonist medetomidine in combination with ketamine. We hypothesized that cardiac arrest with the sevoflurane based anesthesia would provide more neuroprotection than the IP administrated anesthesia.

## Methods

The study was approved by the Animal Care and Experimentation Committee of the Canton of Bern, Switzerland and followed the Swiss national guidelines for the performance of animal experiments. We used 130 male, 10 weeks old Wistar rats (Janvier-Labs, Isle-St-Genest, France) weighting 389 ± 37 g, which were housed in the core animal facilities of the University of Bern with a 12/12 h light/dark cycle at 22°C, with food and water ad libitum.

### Pilot phase

Of the total of 130 rats, 57 were used to test different cardiac arrest times of 6, 7, 7.5, and 8 minutes by either induction of ventricular fibrillation or asystole. In these pilot series we could not establish sustained ROSC after induction of ventricular fibrillation, so we used the potassium/esmolol-induced asystolic CA as described below. After establishing 8 minutes as the target, the remaining 73 rats were included in the study to evaluate the damage and recovery after cardiac arrest.

### Study procedures - overview

The study protocol consisted of induction of anesthesia, surgical preparation, adjustment of mechanical ventilation based on post-surgery blood gas analysis, induction of cardiac arrest for 8 minutes, resuscitation, and behavioral follow-up after successful resuscitation for 1, 2, or 5 days. At the end of follow up, the animals were euthanized and brain tissue was harvested for analyses from animals followed up for 2 and 5 days.

### Allocation

The animals were anesthetized either with sevoflurane/fentanyl (sev/fent) or medetomidine/ketamine (med/ket). Assignment to the different anesthesia groups was not randomized for logistic reasons: the vaporizer was only available at certain days. At these days, the ventilator stayed configured for the vaporizer and no experiments of the other group were conducted and vice versa. Assignment to duration of follow up was decided simultaneously with the anesthesia regime before the experiment, and an even distribution between groups was pursued. No cross-over after assignment was allowed. However, because low overall survival rates, we decided to abandon the day one follow up and to redistribute the remaining animals into the 2 and 5 days follow-up.

### Anesthesia and surgery

Animals in the sevoflurane/fentanyl group (sev/fnt) were placed in an induction chamber with 6% sevoflurane (Sevorane, Abbott Switzerland), with an additional dose of fentanyl (Fentanyl, Janssen-Cilag, Switzerland) 20 μg/kg intraperitoneally (IP). The animals in the medetomidine/ketamine (med/ket) group received IP a mixture of 0.25 mg/kg Medetomidine (Medetor, Virbac Switzerland) and 60 mg/kg ketamine (Ketalar, Pfizer Switzerland). Orotracheal intubation was performed with an angiocath 2.0 mm diameter (Venflon, BD Germany) and the position was confirmed by capnography (Datex S/5 Anaesthesia Monitor, GE, Helsinki, Finland). Animals were ventilated with a pressure-controlled, time-cycled ventilator (KTR-5, Hugo-Sachs Elektronik, March, Germany). The initial ventilator settings were 50 breaths per minutes, 30% oxygen/air mix, an inspiratory pressure of 12 cm H2O, and a PEEP of 3 cm H20. After stabilization (10 minutes), blood gases were analyzed and the inspiratory pressure of the ventilator adapted to achieve normoventilation. To spare blood, no further blood gas analysis was performed before cardiac arrest. The animals were fixed on a surgical heating pad, a thermometer was placed into the esophagus, and the animals were warmed to 36°. Subcutaneous ECG electrodes were placed on standard positions. The right femoral artery was exposed and a PE 50 catheter for blood pressure monitoring and blood sampling was inserted. Another PE 50 catheter for drug administration was inserted into the right jugular vein. All operations were done by either MH or DS. Time from anesthesia induction to start surgery took about 15 minutes in the med/ket group and 10 minutes in the sev/fnt group. The surgical procedure was about 20 minutes in length. Recordings of ECG, arterial blood pressure and temperature were performed with a standard anesthesia monitor (Datex S/5 Anaesthesia Monitor, GE, Helsinki, Finland) and the Wincollect data logger (GE, Helsinki, Finland) at a rate of 100 Hz.

### Cardiac arrest and resuscitation

The rats were paralyzed with 2 mg/kg body weight vecuronium (Norcuron, MSD, Switzerland) via the jugular vein catheter to suppress agonal breathing during the experiment. Cardiac arrest was induced with a mixture of potassium chloride and esmolol (Esmolol-Orpha, OrPha Swiss, Switzerland). The rational for adding esmolol was to lower the total amount and the concentration of the KCl solution, as done for cardioplegia in human cardiac surgery. The final solution had a concentration of 0.2 mmol/ml KCl and 9 mg/ml esmolol. Initially, a dose of 2 ml/kg body weight (BW) was given until cardiac arrest followed, indicated by a drop of the arterial blood pressure below 15 mmHg, which occurred in all animals within 5 seconds. If electrical cardiac activity in the ECG was present, immediately an additional dose of the mixture was added (up to totally 1 ml). The catheter was flushed with 0.2 ml of a 0.02 mmol/ml KCl solution. This lower concentrated KCL flush- solution was used to avoid “auto-resuscitation” which occurs when some spontaneous very weak contractions of the heart, sometimes ensuing about 1–2 minutes after cardiac arrest, increase in strength and frequency. Ventilation was disconnected, and the heating lamp turned off. After 7.5 minutes, the heating lamp was turned on, the ventilator was reconnected, a recruitment maneuver performed (sustained inspiratory pressure of 20 mmH2O), and ventilation resumed with 55 breaths per minute and 100% oxygen. At 8 minutes, manual metronome guided chest compression with 2 fingers at a rate of 220/min were started. The chest compression were made always by the same two operators, compression depth was about 25 percent of the anterior–posterior diameter of the chest with complete recoil, and targeted to achieve a mean artery pressure (together with adrenaline) of 50 – 55 mmHg. Diluted adrenaline 15 μg/kg (Adrenalin Bichsel, Switzerland) and 0.2 ml of a diluted calcium solution containing 0.1125 mmol Ca/ml (Calcium-Sandoz, Sandoz, Switzerland) were injected at the beginning of resuscitation maneuvers, separated by 0.1 ml of normal saline. If ROSC was not achieved within 60 seconds, adrenaline was repeated with 5 μg/kg every 30 seconds until ROSC. Calcium was repeated after 2 minutes of resuscitation. ROSC was defined as regular cardiac activity > 200 beats per minute with a mean arterial pressure of ≥ 60 mmHg sustaining for at least 20 seconds (one monitor length). If ROSC was not achieved within 4 minutes, no further resuscitation attempts were made.

After successful resuscitation, ventilation was increased to 70 breaths per minute, with inspiratory pressures of 18–20 mmH2O, and the rats received 50 mg/kg BW ampicillin (Clamoxyl, GlaxoSmithKline, Switzerland) for perioperative antibiotic prophylaxis and 20 μg/kg BW buprenorphine (Temgesic, Reckitt Benckiser, Switzerland) as pain relieve intramuscularly. The animals in the sev/fnt group received 0.6 to 0.8% sevoflurane once hemodynamics were stable, the concentration was targeted accordingly to avoid excessive hypertension and ventilator dyssynchronicity. Blood for arterial blood gases were drawn at ROSC +5 min and ROSC + 15 min. The catheters were withdrawn immediately afterwards, the wounds closed and the mechanical ventilation weaned within the following 15 minutes. The still comatose rats were placed on a heating mattress, without additional oxygen. After 4 hours, the rats were returned into a single cage with access to water and food ad libitum.

### Follow-up after resuscitation

Animals were followed according the Animal Care and Experimentation Committee of the Canton of Bern for general health, including daily weight measurement with an electronic balance.

Neurological functions were assessed daily from day 1 to day 5 with an established wide-ranging neuroscore (NDS) [14]. The NDS evaluates general behavior and respiration (maximum, 40 points), cranial nerves function (maximum, 20 points), sensitivity to tactile stimuli (maximum, 10 points), motor function (maximum, 10 points), and coordination (maximum, 20 points).

To assess gross locomotor abilities, a modified simple vertical pole test [15] was used. The test scores the capability of the animals to move along a horizontal wooden spar of 1.5 cm width, followed by stepwise elevation of the bar to 45° to 90°, with the animal still on it. Inability to move along the horizontal spar (0°) scored 3, keeping at 90° scored 0.

To evaluate sensorimotor integration, the Tape Removal Test was used [16]. This test measures the time needed to successfully remove a standard (10×12 mm) adhesive tape attached to the front paw of the animals (maximum 120 seconds).

To determine the general activity level, the Open Field task was used [17]. Assessment took place in a square (0.75 × 0.75 m), wooden box, with the floor divided by marks in 25 squares. The animals were placed in the arena and allowed to freely move for 4 minutes while being recorded by an overhead camera. Normal behavior of rats consists of moving around and exploring the arena, including doing rears to investigate the surroundings. Accordingly, we analyzed footage by hand counting the squared crossed (with at least three paws) and rears.

### Euthanasia and histology

In order to estimate neuronal damage consecutive to cardiac arrest and resuscitation, animals were euthanized at day 1, day 2 or day 5 with an overdose of pentobarbital intraperitoneally. Then the animal was transcardially perfused via the left ventricle with 150 ml of buffered formalin 4% in PBS (Hospital pharmacy Inselspital Bern, Switzerland). A vertical midline incision extending from the level of the cervical-cranial junction to the tip of the nose was performed with scissors. The skull edges were retracted with forceps so that the exposed brain could be removed intact. Brains were post-fixed in buffered formalin 4% in PBS for 24 hours at 4°C. The brains were then transferred to an 18% sucrose solution in PBS at 4°C until further processing.

Histological cryo-sections were obtained by cutting the brain (45 μm thick coronal sections) using a Leica CM 1850 cryostat, after snap freezing of the brain in methylbutane pre-cooled at -80°C. Injury to the hippocampus was determined by histomorphometry on 4 sections of different hippocampal regions (from rostral to caudal, therefor examining mostly the dorsal part of the cornus ammoni 1 (CA1 segment) stained with cresyl violet [18]. Histological sections were scanned at a resolution of 2700 dpi using a dia scanner (Polaroid SprintScan 35 Plus). Atrophy of the cornus ammoni 1 cell layer, resulting from neuronal loss was determined using the software ImageJ 1.45 l (National Institutes of Health, USA, http://imagej.nih.gov/ij). For this aim, the area of the CA1 subfield stained by cresyl violet was determined. To correct for differences in the length of the CA1 between sections, normalization was performed by dividing the calculated surface by the length of the structure, determined at its border proximal to the dentate gyrus, and expressed as cell layer ratio (area/length). Furthermore, the percentage of cells with apoptotic morphology (pyknotic nucleus, shrunken appearance) was estimated in the CA1 region of the same 4 sections by visual observation under a bright-field microscope, and reported as % pyknotic cells. Because the rostral/septal portion of the hippocampus is especially vulnerable, the two rostral/septal and the two caudal/temporal segments were analyzed separately. The correlation between atrophy and apoptosis was estimated with the Pearson’s correlation coefficient (see below).

All histopathological evaluations were performed by the same investigator blinded to the clinical and treatment data of the respective animal.

### Cerebrospinal fluid assessment

Cerebrospinal fluid was obtained by puncture of the cisterna magna with a 26 gauge injection needle via the suboccipital access after the pentobarbital injection was given. Between 50 and 80 μl of CSF could be harvested, immediately centrifuged and then stored at -80°C until further examination. Measurement of cytokines/chemokines concentrations in the undiluted CSF samples was performed using the suspension array technology (http://www.luminexcorp.com), using a Millipore MILLIPLEX MAP Rat Cytokine/Chemokine Magnetic Bead Panel (Millipore AG, Zug, Switzerland). Quantification was based on the use of standard curves for each analyte. Concentrations of cytokines were calculated using Bio-Plex Manager Software 4.1 (Bio-Rad Laboratories) with a 5-parameter logistic curve-fitting method. The cytokines/chemokines measured were: interleukin 6 (IL-6, lower limit of detection 30.7 pg/ml); TNF-α (lower limit of detection 1.9 pg/ml); monocyte chemoattractant protein-1 (MCP-1, lower limit of detection 9.0 pg/ml) and macrophage inflammatory protein-1α (MIP-1α, lower limit of detection 0.8 pg/ml).

### Statistics

Because of the small numbers in the groups, non-normal distribution was assumed. Data are presented as medians and [IQR]. Proportions of survival were analyzed with the Fisher’s exact test. Baseline values were compared using Mann–Whitney *U* test. Repeated measurements were assessed with Friedman test. Either significant or insignificant Friedman tests in both groups were interpreted as indicating no differences between groups, whereas a significant test in one together with an insignificant test in the other group as indicating a difference between groups. In order to have the same number of general health and neurologic test assessments from all animals, primarily only baseline, first and last days’ assessment were used for the statistical analysis. In order to evaluate potential changes between day 2 and day 5 in the group with the longest survival, a separate Friedman Test was performed in this group of animals including assessment from days 2 to 5. Pre-cardiac arrest blood gases were not included in the statistical analysis because the ventilator settings were adapted afterwards but the effect on blood gases not measured thereafter. The non-parametric Pearson correlation test was used to estimate the correlation between both histomorphometric methods (amount of cells with apoptotic morphology resulting in CA1 atrophy and normalized surface). A p < 0.05 was considered as significant. The calculations were performed using SigmaPlot/SigmaStat 12.5 (Systat Software GmbH, Erkrath, Germany).

## Results and discussion

**Figure 1:**
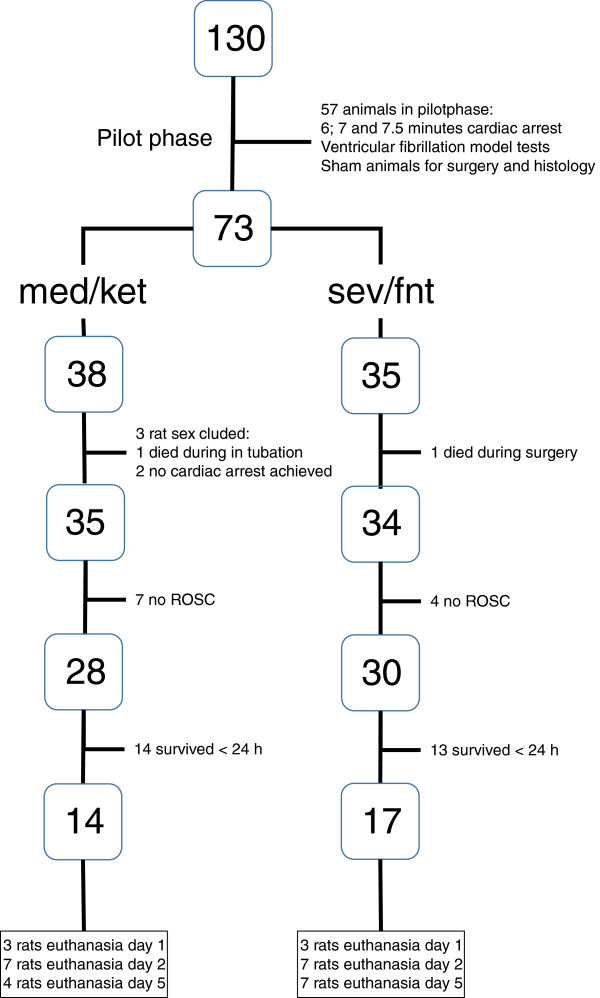
**Flow chart of the number of animals assigned into the different groups.**

**Figure 2:**
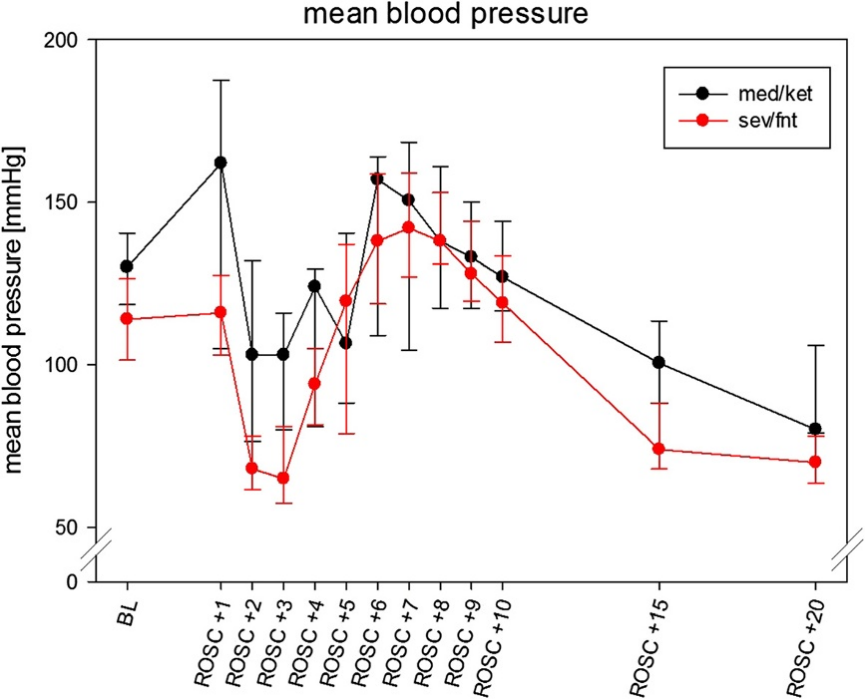
**Mean blood pressure at different time points.** Variation of blood pressure is similar in both groups. Data are shown as means and [IQR].

From the 73 animals that entered the experiment, cardiac arrest was induced successfully in 69. Return of spontaneous circulation (ROSC) was achieved in 58 (84%) and of those with ROSC 31 (53%) survived for more than 24 hours (Figure 1). The 27 animals which survived < 24 hours almost all died within the first 6 hours, after presenting an increasing rapid shallow breathing pattern, indicating atelectasis. There was no difference between the anesthesia regimens in success to achieve cardiac arrest and ROSC (med/ket 28 of 35 vs sev/fnt 30 of 34, p = 0.51), in overall survival beyond 24 hours (14 of 35 in med/ket, 17 of 34 in sev/fent p = 0.47), or in survival beyond 24 hrs after ROSC (17 of 28 in med/ket, 17 of 30 in sev/fent, p = 0.80; Figure 1).The amount of the potassium chloride/esmolol mixture did not differ in both groups (sev/fnt 1.0 ml [IQR 0.88 - 1.0] vs 1.0 ml [IQR 0.7 - 1.0] in med/ket, p = 0.25). The time to achieve ROSC was shorter in the sev/fnt group (75 [IQR 60 – 90] sec vs 91 [IQR 90 – 140] sec, p = 0.02), and there was no significant difference between the groups in the amount of adrenaline needed (sev/fnt 8.6 μg [IQR 7.0 - 11.0] vs 9.0 [8.0 - 11.2] in med/ket, p = 0.50). Blood pressure varied during the first 20 minutes after ROSC (p < 0.01) similarly in the two groups, although the sev/fnt group had a more severe initial hypotension early after ROSC (Figure 2).

**Figure 3:**
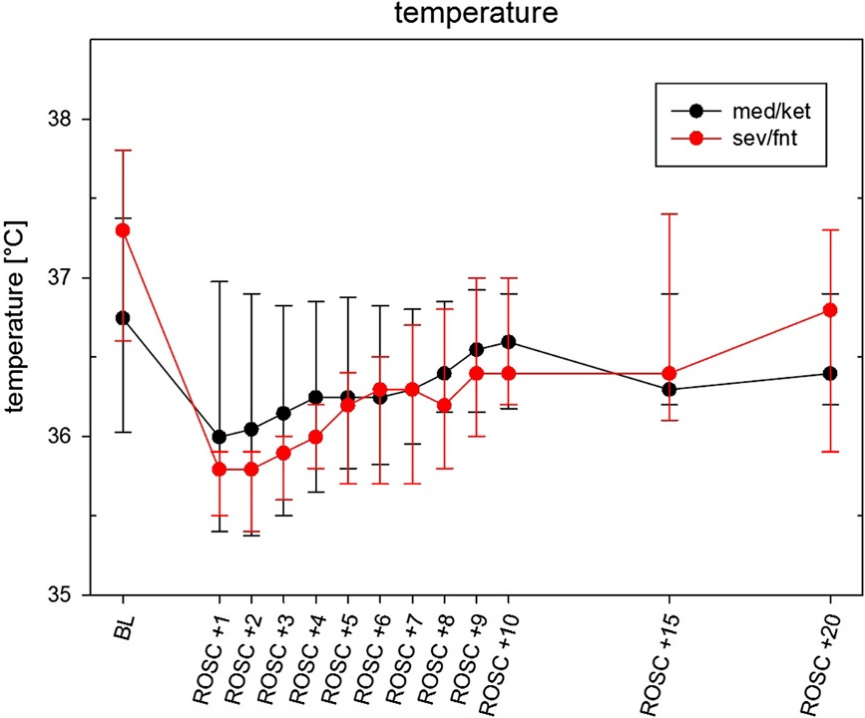
**Temperature at different time points.** Friedmans’s test within each anesthesia group is significant (both p < 0.01), but at no time there is a significant difference between both groups. Data are shown as means and [IQR].

After ROSC both groups were briefly hypothermic, but normalized the body temperature within 20 minutes after ROSC (Figure 3). In the first blood gas analysis (BGA) after insertion of the arterial line, before cardiac arrest and adjustments in ventilation, animals in the sev/fnt group had respiratory alkalosis (pH 7.56 [IQR 7.50 – 7.60] vs 7.42 [7.37 – 7.46] in med/ket, p < 0.01; pCO2 26.0 [22.5 – 31.5] mmHg in sev/fnt vs. 39.5 [32.8 – 48.0] mmHg in med/ket, p < 0.01; base excess 2.1 [1.3 – 2.7] in sev/fnt vs 0.2 [-2.0 – 2.6] in med/ket, p = 0.05), increased blood lactate (1.7 [1.5 – 2.0] mmol/l in sev/fnt vs. 0.7 [0.6 – 0.9] mmol/l in med/ket, p < 0.01), and higher pO2 values (190 [163 – 229] mmHg in sev/fnt vs. 124 [93 – 154] mmHg in med/ket, p < 0.01) compared to the med/ket group. To spare blood, no further BGAs were drawn after ventilator adjustments and before CA, we assumed identical metabolic and similar respiratory status (because low pCO2 with accompanying alkalosis shifts the ubiquitous lactate-pyruvate dehydrogenase toward the lactate, this increases the measured lactate, but is per definition no lactic acidosis, and no metabolic derangement). Both groups recovered similarly from metabolic and respiratory acidosis between ROSC +5 min and +15 min (Table 1). Glucose was significantly higher at baseline (18.0 [IQR 15.0 – 19.4] mmol/l in med/ket vs. 10.1 [9.5– 11.7] mmol/l in sev/fnt, p < 0.01) and all later time points in the med/ket group (Table 1).

**Table 1:** **Values of the arterial blood gas analysis 5 and 15 minutes after return of spontaneous circulation (median [IQR])**

		Medetomidine/ketamine	Sevoflurane/fentanyl	p between groups Mann–Whitney test
pH	5 min	7.03 [IQR 6.93 – 7.09]	7.03 [IQR 6.95 – 7.15]	p = 0.44
	15 min	7.31 [IQR 7.24 – 7.34]	7.21 [IQR 7.07 – 7.32]	
	p within groups Wilcoxon test	p < 0.01	p < 0.01	
Base excess	5 min	-18.2 [IQR -20.1 – -15.6]	-16.3 [IQR -19.1 – -14.3]	p = 0.18
	15 min	-9.6 [IQR -12.0 – -7.6]	-10.0 [IQR -14.4 – -8.1]	
	p within groups Wilcoxon test	p < 0.01	p < 0.01	
pCO_2_ [mmHg]	5 min	54.0 [IQR 39.9 – 64.3]	49.0 [IQR 42.5 – 67.5]	p = 0.83
	15 min	33.0 [IQR 29.8 – 38.0]	43.0 [IQR 33.0 – 53.0]	
	p within groups Wilcoxon test	p < 0.01	p < 0.01	
pO_2_ [mmHg]	5 min	123 [IQR 113 – 1137]	110 [IQR 97 – 124]	p = 0.15
	15 min	141 [IQR 99 – 157]	92 [IQR 79 – 117]	
	p within groups Wilcoxon test	p = 0.39	p = 0.01*	
Lactate [mmol/l]	5 min	8.3 [IQR 6.9 – 10.7]	7.7 [IQR 7.4 – 9.3]	p = 0.86
	15 min	4.0 [IQR 3.1 – 6.7]	4.5 [IQR 3.8 – 4.7]	
	p within groups Wilcoxon test	p < 0.01	p < 0.01	
Glucose [mmol/l]	5 min	22.6 [IQR 20.4 – 25.3]	14.9 [IQR 13.3 – 16.4]	p < 0.01*
	15 min	21.9 [IQR 19.6 – 23.7]	13.1 [IQR 12.2 – 14.5]	p < 0.01*
	p within groups Wilcoxon test	p = 0.08	p = 0.01*	

Mixed respiratory and metabolic acidosis are even distributed after cardiac arrest and corrects similarly in both groups. pO_2_ is lower after 15 minutes in the sev/fnt group (significant decline from the 5 minutes value, compared to the med/ket group. Glucose values are always higher in the med/ket group due to the medetomedine (details see text) * indicate significant differences between the anesthesia groups.

Animals lost weight initially, but recovered similarly in both groups. The Neuro Deficit Score, Vertical Pole Test and Tape Removal Test demonstrated a decreased performance from baseline with recovery at the last assessment day without differences between anesthetic groups. In those animals surviving 5 days, no changes occurred between day 2 and day 5. In the open field, in both anesthetic groups the numbers of rears decreased significantly after cardiac arrest without recovery after day 2. The numbers of squares crossed was similar if assessed during baseline, day 1 and last assessment in both groups. In those animals surviving for 5 days the assessment of numbers of squares crossed after day 2 demonstrated a reduced mobility in the medetomidine/ketamine group (p = 0.04; Table 2).

**Table 2:** **Results of the general health and neurologic tests**

		Medetomidine/ketamine	Sevoflurane/fentanyl	p between groups Mann–Whitney test
Weight [g]	BL	385 [IQR 357 – 413]	380 [IQR 360 – 426]	p = 0.89
	day 1	375 [IQR 353 – 415]	367 [IQR 343 – 420]	
	day 2	360 [IQR 322 – 400]	355 [IQR 342 – 387]	
	day 3	359 [IQR 322 – 413]	359 [IQR 348 – 360]	
	day 4	369 [IQR 320 – 417]	349 [IQR 343 – 351]	
	day 5	370 [IQR 325 – 421]	350 [IQR 319 – 360]	
Friedman & (BL, day 1 and last day, all animals)		p < 0.01	p < 0.01	
Friedman^%^ (day 2–5, only animals surviving all days)		p = 0.14	p = 0.58	
NDS	BL	0 [IQR 0 – 0]	0 [IQR 0 – 0]	p = 0.34
	day 1	10 [IQR 5 – 14]	5 [IQR 3 – 10]	
	day 2	5 [IQR 0 – 5]	0 [IQR 0 – 5]	
	day 3	0 [IQR 0 – 0]	0 [IQR 0 – 15]	
	day 4	0 [IQR 0 – 0]	0 [IQR 0 – 15]	
	day 5	0 [IQR 0 – 0]	0 [IQR 0 – 15]	
Friedman & (BL, day 1 and last day, all animals)		p < 0.01	p < 0.01	
Friedman^%^ (day 2–5, only animals surviving all days)		p = 0.93	p = 0.07	
OFT [n]	BL	94 [IQR 75 – 110]	91 [IQR 64 – 114]	p = 0.89
	day 1	107 [IQR 26 – 147]	102 [IQR 52 – 163]	
	day 2	106 [IQR 55 – 130]	114 [IQR 81 – 158]	
	day 3	35 [IQR 17 – 93]	90 [IQR 63 – 135]	
	day 4	35 [IQR 9 – 66]	42 [IQR 13 – 148]	
	day 5	20 [IQR 11 – 33]	66 [IQR 23 – 138]	
Friedman & (BL, day 1 and last day, all animals)		p = 0.23	p = 0.76	
Friedman^%^ (day 2–5, only animals surviving all days)		p = 0.04	p = 0.73	
rears [n]	BL	23 [IQR 17 – 29]	19 [IQR 16 – 25]	p = 0.21
	day 1	2 [IQR 0 – 13]	5 [IQR 1 – 12]	
	day 2	12 [IQR 1 – 14]	11 [IQR 4 – 19]	
	day 3	7 [IQR 3 – 11]	6 [IQR 3 – 11]	
	day 4	6 [IQR 4 – 8]	5 [IQR 0 – 13]	
	day 5	5 [IQR 3 – 7]	4 [IQR 0 – 13]	
Friedman & (BL, day 1 and last day, all animals)		p < 0.01	p = 0.02	
Friedman^%^ (day 2–5, only animals surviving all days)		p = 0.21	p = 0.18	
VPT	BL	0 [IQR 0 – 0]	0 [IQR 0 – 0]	p = 0.70
	day 1	2 [IQR 1 – 2]	2 [IQR 2 – 2]	
	day 2	1 [IQR 1 – 2]	1 [IQR 1 – 2]	
	day 3	0 [IQR 0 – 1]	1 [IQR 1 – 1]	
	day 4	0 [IQR 0 – 0]	0 [IQR 0 – 2.0]	
	day 5	0 [IQR 0 – 1]	0 [IQR 0 – 1.0]	
Friedman & (BL, day 1 and last day, all animals)		p < 0.01	p < 0.01	
Friedman^%^ (day 2–5, only animals surviving all days)		p = 0.16	p = 0.43	
TRT [sec]	BL	18.5 [IQR 11.5 – 23.5]	11.0 [IQR 9.5– 19.5]	p = 0.12
	day 1	120 [IQR 120 – 120]	120 [IQR 93.5 – 120]	
	day 2	55.0 [IQR 15.0 – 120]	48.0 [IQR 29.5 – 120]	
	day 3	32.0 [IQR 11.8 – 95]	62.0 [IQR 19.0– 120]	
	day 4	46.0 [IQR 21.0 – 102.5]	28.0 [IQR 19.0– 120]	
	day 5	24.0 [IQR 12.3 – 96.5]	36.0 [IQR 18.0– 112]	
Friedman & (BL, day 1 and last day, all animals)		p < 0.01	p < 0.01	
Friedman^%^ (day 2–5, only animals surviving all days)		p = 0.65	p = 0.80	

Friedman test including baseline, first and last day assessments (all animals)&, and including assessment from day 2 to 5, respectively (only animals with longest survival)^%^. No significant differences could be found between both anesthesia groups at baseline, and global (BL, day 1 and last day) Friedman’s tests (time effect) did not diverge. The only difference found is a reduction in the numbers of squares crossed in the open field test during the recovery phase after day 2 in the med/ket group, compared to the sev/fnt group, in which the animals remained mobile.

NDS: Neuro Deficit Score; OFT: Open Field Test (numbers of squares crossed in the open field); rears: number of rears in the open field; VPT: Vertical Pole Test, TRT: Tape Removal Test.

**Figure 4:**
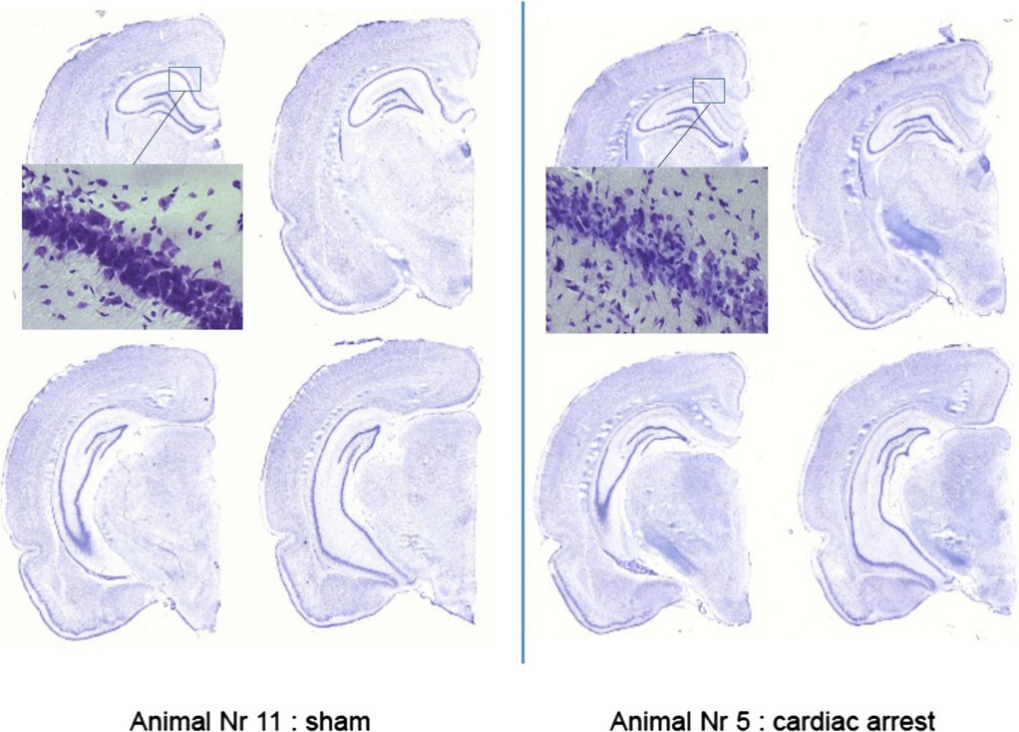
**Example of injury to the hippocampus (cresyl violet staining).** Left: sham animal, right: 8 minutes cardiac arrest. The 4 different slides of one hemisphere are showing the different sections of the CA1 segment of the hippocampus while moving from rostral to caudal (starting up-left, up-right, down-left and down-right). Delineating the border of the CA1 segments is difficult in the caudal sections because of the curvature of the hippocampus. The inlays demonstrate the shrunken and pyknotic neurons, resulting in a diminished cell layer of CA1 in the cardiac arrest animal (400x). Histomorphometric analysis of the CA1 segment was performed by 1. cell count and 2. automated surface area calculation (details see Methods). The sham animal was operated in the pilot phase, and received complete surgery under sev/fnt anesthesia, but was not subjected to cardiac arrest.

Histologic examinations (cell count) and histomorphometry (cell layer area) demonstrated a highly significant inverse correlation between both methods (p < 0.01, correlation coefficient < -0.8), especially in the two most rostral sections of the hippocampus CA1 segment, where most of the damage should be expected [19]. There was no apoptosis on day 2 in either group. On day 5, significantly more cells with pyknotic nuclei could be found in the rostral and caudal parts of the animals in the sevoflurane group (Table 3). This difference did not translate into variable cell layer thickness on day 5 (Table 3), although the cell layer stratum decreased between day 2 to day 5 (see Table 4 and Figure 4).

**Table 3:** **Results of the histology analysis of the rostral and caudal sections of the CA1 segment of the hippocampus on day 5**

			Day 5			
	Cell layer (ratio area/length)			Pyknotic cells %		
	Med/ket	Sev/fnt	p	Med/ket	Sev/fnt	p
CA1 rostral	0.058 [0.055 – 0.064]	0.055 [0.030 – 0.065]	0.65	19 [7 – 28]	85 [60 – 88]	0.01^*^
CA1 caudal	0.093 [0.081 – 0.108]	0.110 [0.090 – 0.110]	0.32	4 [1 – 21]	33 [27 – 63]	0.02^*^

On day 2, there were virtually no cells with pyknotic nuclei. Data are shown as medians and interquartile range in square brackets [], *denotes significant differences. The “thickness” of the cell layer was normalized as ratio of the area and the length of the examined part (see Methods section).

**Table 4:** **time course of the decrease of the CA1 cell layer area as a marker of atrophy**

	Cell layer (ratio area/length)					
		Med/ket			Sev/fnt	
	Day 2	Day 5	p	Day 2	Day 5	p
CA1 rostral	0.085 [0.070 – 0.090]	0.058 [0.055 – 0.064]	<0.01^*^	0.075 [0.070 – 0.080]	0.055 [0.030 – 0.065]	<0.01^*^
CA1 caudal	0.125 [0.120 – 0.135]	0.093 [0.081 – 0.108]	< 0.01^*^	0.120 [0.105 – 0.123]	0.110 [0.090 – 0.110]	0.30

Data are shown as medians and interquartile range in square brackets [], *denotes significant differences. The “thickness” of the cell layer was normalized as ratio of the area and the length of the examined part (see Methods section).

The levels of cytokines in the CSF were below the detection threshold for IL-6, TNF-α and MIP-1α. Only MCP-1 could be detected. On Day 2 MCP-1 was 69.1 pg/ml [IQR 57.7 - 90.7] in the sev/fnt group and 72.1 pg/ml [IQR 45.8 – 101.4] in the med/ket group (p = 1.0) On day 5 MCP-1 was higher in the sev/fnt group than in the med/ket group (101.4 pg/ml [IQR 57.7 - 179.7] vs. 39.6 pg/ml [IQR 33.3 - 87.5], respectively; p = 0.04). vs). There was no difference in MCP-1 between Day 2 and Day 5 in either group.

Experimental cardiac arrest and cardiopulmonary resuscitation models help to elucidate the mechanisms of brain injury and to develop therapeutic strategies. The role of anesthesia in these models has received little attention despite the different neuroprotective properties of various anesthetics. The cerebral effects of anesthetic agents after cardiac arrest are clinically relevant since patients who remain comatose after return of spontaneous circulation are routinely treated with sedative and anesthetic agents in the ICU, although not with the combinations tested in this study. We found in our rodent model of cardiac arrest that the choice of either medetomidine and ketamine or sevoflurane and fentanyl did not modify the overall clinical and behavioral effects of post cardiac arrest brain damage. Nevertheless, the use of medetomidine and ketamine compared to sevoflurane and fentanyl decreased apoptosis in the hippocampus and decreased the concentration of the inflammatory cytokine MCP-1 in the CSF on day 5 post cardiac arrest.

Sevoflurane has been associated with cerebral neuroprotective effects in experimental myocardial ischemia and focal cerebral ischemia models [20, 21], as well as in human cardiac surgery [22]. In contrast, in piglet cardiac arrest adding sevoflurane to hypothermia provided no additional neuroprotection [23]. We found more apoptosis with sevoflurane and fentanyl than with medetomidine and ketamine. This suggests that the effects of sevoflurane may depend on the model of ischemia. Also the timing of exposure to sevoflurane may be relevant. In a cell culture model of hypoxia, sevoflurane was either protective, harmful or had no effect depending on the timing and duration of exposure [24].

An alternative explanation is that the α-agonist medetomidine as well as the NDMA-blocker ketamine were neuroprotective. Such protective effects have been observed in focal ischemia model of stroke when added to halothane [25] or compared to an glutamate receptor antagonist [26] or different other compounds and anesthetics [27].

Increased hippocampal apoptosis and behavioral impairment after circulatory arrest has been reported by others in asphyxia-induced cardiac arrest models [14, 28]. The lack of behavioral impairment in our model despite comparable hippocampal histological alterations and longer circulatory arrest time is likely to be related to lack of preceding asphyxia in our model. Also, our low-flow time was shorter than the low-flow times reported by others.

The shorter low-flow time in the sev/fnt group did not translate to a better outcome. The explanation of the shorter cardio-pulmonary resuscitation time may be better preserved cardiac function after cardiac arrest [29]. Addition of sevoflurane to a pentobarbital anesthesia in a similar CA model resulted in a better cardiac function up to 24 hours after CA, but had no beneficial effect on survival.

The distance travelled in the Open Field Test (counted by the numbers of squares crossed) was similar in both groups if regarded to all time points. A small, albeit significant difference appeared after day 2 in the recovery phase, where the animals in the sev/fnt remained mobile compared to the animals in the med/ket group. In view of the reduced delayed hippocampal apoptosis in the med/ket group a cautionary explication would be the preserved memory in these animals, so they learned and retained the environment and expressed less exploratory behavior. Not in line with this explanation is the missing difference in the number of rears, and similar results in the other clinical tests, so this finding is rather unspecific.

Regarding the inflammatory markers in the CSF, all markers except the proinflammatory marker MCP-1 were below the detection threshold and even MCP-1 was very low. Accordingly, the relevance of increased MCP-1 in sev/fnt at day 5 should be interpreted with caution. Monocyte chemoattractant protein-1 MCP-1 [also referred as chemokine (C-C motif) ligand 2 (CCL2)] is expressed by resident CNS glial cells in response to virtually any insults (trauma, infection, hypoxia) and is involved in the recruitment of inflammatory cells in the CNS [30]. Increased levels of various CSF biomarkers after cardiac arrest have been reported in experimental cardiac arrest models [31] and after cardiac arrest in patients. The neurofilament light protein (NFL), total tau (t-tau) and YKL-40 were strongly associated with outcome [32]. The lack of more severe signs of neuroinflammation in our model may be related to the lack of hypoxia or low cerebral blood flow before the cardiac arrest and to the short duration of cardiopulmonary resuscitation after the arrest [33].

The higher glucose concentration in the med/ket group was likely due to the α-agonist medetomidine, which interferes with insulin secretion [34] or due to a higher stress response to intraperitoneal injection as compared to inhalational anesthesia. Although hyperglycemia is considered detrimental after cardiac arrest [35], hyperglycemia in the med/ket group did not translate into an overall worse outcome in our model.

The initial aBGA demonstrated a respiratory alkalosis with consecutive higher lactate values in the sev/fnt group, despite the same initial ventilator settings. This is due to the better muscle relaxant properties of the sevoflurane/fentanyl combination [36] compared to medetomidine/ketamine, resulting in smoother ventilation and less animal-ventilator asynchrony. We assumed normal aBGA in both groups after adaptation of the ventilation because after resuscitation the values were similar. We considered the maintenance of blood volume as a priority against the verification of full normalization of blood gases, therefore the pre-correction aBGA was not included in the time series analysis.

The 27 animals surviving less than 24 hours died in the early post-resuscitation phase, after presenting a rapid shallow breathing pattern. This indicates atelectasis, but could also be a sign of progressive cardiac failure and cardiovascular collapse. In later experiments, performed after finishing this series, we increased ventilation time after ROSC for 4 hours and by doing so, we were able to eliminate early death after ROSC.

Our study has limitations. The assignment into the different groups was not randomized for logistic reasons. Since the animals were assigned to the different duration of follow-up before the experiment, a potential bias of less severe injury in the longer follow-up could be avoided.

Another limitation is the use of two anesthetic regimes rather than two anesthetics, but this cannot be avoided. So we cannot assure equipotent doses of anesthetics, the same is true for pharmacokinetic and pharmacodynamic effects. The effect of med/ket seemed to last longer than sev/fnt because post resuscitation we never needed any re-dosing of med/ket, but all animals in the sev/fnt group received sevoflurane at a low dose of 0.6 to 0.8% to avoid excessive hypertension and fighting against the ventilator. The slightly lower blood pressure during the experiment in the sev/fnt group is not significantly different than in the med/ket group. We cannot exclude the nonsignificant difference in blood-pressure contributes to the difference seen in the results, but this seems rather unlikely.

The study was not designed to quantify neuroprotective effects of an anesthetic or an anesthetic combination after cardiac arrest, so the difference in the apoptosis rate is the sum effect of both anesthetic combinations on possible precondition, hemodynamic effects (during and post-resuscitation), low-flow time and possible postconditioning.

Post resuscitation care was minimal and was for example not controlled for body temperature (beyond 20 minutes), which is a further limitation for generability in this study. A longer post resuscitation period would have posed a different problem with the anesthetic regimes for re-dosing of med/ket would be more difficult than with sevoflurane.

The overall survival of 53% beyond 24 hrs indicates that our model is quite severe, and it is possible that the lack of histology and CSF analysis in the early dying animals missed some of the typical changes reported by others. The small number of animals per group is another limitation.

The lack of consistent differences in the functional tests might be explained by insensitivity of the behavioral tests for detecting hippocampal CA1 related dysfunction, where learning and memory abnormalities can be expected [37]. If the use of more elaborate tests (Morris water maze, novel object recognition and location memory) would have changed the results remains speculative; the small number of animals might also explain the absence of a detectable difference in the functional tests.

Extrapolation to clinical cardiac arrest is limited, since we used these anesthetics already before the cardiac arrest. The impact of these anesthetic agents, when administered first after the ROSC should be addressed in future studies.

Although our experiment explored neurologic function in a model of cardiac arrest, the results have implication for other animal models of neurological insults as stroke or traumatic brain injury: the choice of anesthetics should be taken into account in the design and interpretation. Based on our study we propose, depending on the design and research question, to introduce a second control group with a different anesthetic regime to rule out the possibility that the results are biased by the effects and side effects of the anesthetic regime.

## Conclusion

No difference in neurological function could be detected on day 5 post cardiac arrest between the anesthetic groups in our rat cardiac arrest resuscitation model. Sevoflurane/fentanyl seem to have no neuroprotective effect as compared to ketamine/medetomidine, based on the lower apoptosis rate. The more prominent apoptosis with sevoflurane/fentanyl indicates that the method of anesthesia should be considered in interpreting the results of experimental models of cardio-pulmonary resuscitation.

## Abbreviations

CA1: Cornus ammonis segment 1
CSF: Cerebrospinal fluid
IL-6: Interleukin 6
IP: Intraperitoneal
MCP-1: Monocyte chemotactic protein 1
med/ket: Medetomidine/ketamine
MIP-1α: macrophage inflammatory protein 1 alpha
nds: Neuro deficit score
oft: Open field test
ROSC: Return of spontaneous circulation
sev/fent: Sevoflurane/fentanyl
TNF-α: Tumor necrosis factor alpha
trt: Tape removal test.
